# Transcriptional regulation and nuclear reprogramming: roles of nuclear actin and actin-binding proteins

**DOI:** 10.1007/s00018-012-1235-7

**Published:** 2012-12-29

**Authors:** Kei Miyamoto, J. B. Gurdon

**Affiliations:** The Wellcome Trust/Cancer Research UK Gurdon Institute, The Henry Wellcome Building of Cancer and Developmental Biology, University of Cambridge, Tennis Court Road, Cambridge, CB2 1QN UK

**Keywords:** Transcriptional activation, Nuclear actin, Transcriptional reprogramming, Nuclear actin-binding protein, Gene silencing, Chromatin remodeling

## Abstract

Proper regulation of transcription is essential for cells to acquire and maintain cell identity. Transcriptional activation plays a central role in gene regulation and can be modulated by introducing transcriptional activators such as transcription factors. Activators act on their specific target genes to induce transcription. Reprogramming experiments have revealed that as cells become differentiated, some genes are highly silenced and even introduction of activators that target these silenced genes does not induce transcription. This can be explained by chromatin-based repression that restricts access of transcriptional activators to silenced genes. Transcriptional activation from these genes can be accomplished by opening chromatin, in addition to providing activators. Once a de novo transcription network is established, cells are differentiated or reprogrammed to a new cell type. Emerging evidence suggests that actin in the nucleus (nuclear actin) and nuclear actin-binding proteins are implicated in these transcriptional regulatory processes. This review summarizes roles of nuclear actin and actin-binding proteins in transcriptional regulation. We also discuss possible functions of nuclear actin during reprogramming in the context of transcription and chromatin remodeling.

## Introduction

Regulation of gene expression is critically important for all living organisms. Gene expression is spatially and temporally regulated during development. For example, genes necessary for brain formation need to be expressed in the brain progenitor cells in development. If those genes are not properly expressed in progenitor cells or expressed in unrelated cells, normal brain formation is impaired. Proper gene expression is controlled by transcriptional activators. Activators, such as transcription factors and chromatin remodelers, directly act on genes to allow transcription by RNA polymerases. Each activator has distinct target DNA sequences or sites, thereby enabling gene-specific transcriptional activation. In general, different cell types contain different activators to comprise their own gene expression networks. Due to these transcriptional regulations, specialized cells remain in the same lineage and do not switch to unrelated cell lineages.

Nuclear reprogramming enables cells to reset transcriptional patterns of specialized cells and establish those of embryonic cells [1]. Reprogramming was first demonstrated by nuclear transfer experiments, where differentiated somatic cell nuclei were transferred to enucleated eggs and the reconstructed cloned embryos then developed to term [2, 3]. Successful reprogramming of transcription programs in cloned embryos has been shown using many different cell types, suggesting that the transcriptional programs of any cell type can be modified towards another cell type. This concept has been further reinforced by the success of factor-mediated reprogramming, creating induced pluripotent stem (iPS) cells [4–6] and transdifferentiation by overexpression of transcription factors [7–9]. During iPS cell production, key transcription factors for pluripotency are overexpressed in somatic cells and establish a new transcription network resembling or almost identical to embryonic stem (ES) cells in the reprogrammed cells. Similarly, overexpression of tissue-specific transcription factors triggers transdifferentiation [9]. Reprogramming experiments thus tell us (1) the importance of transcriptional activators for cell differentiation/dedifferentiation and (2) that gene expression can be altered even in highly specialized cells.

Increasing evidence suggests that actin in the nucleus (nuclear actin) and nuclear actin-binding proteins (ABPs) play an important role in transcriptional activation and transcription [10–12]. Actin has been known as a major component of the cytoskeleton and as a key player in many cellular processes including cell migration, division, and shaping. Actin continuously changes its polymerized states, at least in the cytoplasm; monomeric actin (G-actin) polymerizes at the barbed end to form filamentous actin (F-actin). Significant amounts of actin are also found in the nucleus [13]. Nuclear actin has been identified as an important component of transcriptional machineries and chromatin remodeling complexes. Similarly, numerous actin-binding proteins are present in the nucleus [14] and implicated in transcription and chromatin remodeling [15, 16]. In addition, recent studies indicate that nuclear actin and actin-binding proteins play vital roles in transcriptional activation during cell differentiation and reprogramming [17–19].

This review describes our current knowledge about mechanisms of transcriptional activation and transcriptional reprogramming, followed by discussion of roles of nuclear actin and actin-binding proteins in these cellular events.

## Transcriptional reprogramming

**Fig. 1:**
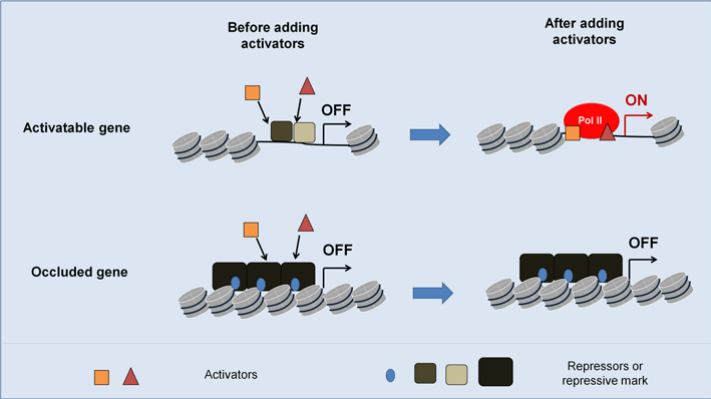
Silenced genes can be classified into two categories (activatable and occluded genes) depending on the resistance to transcriptional activation. After the addition of transcriptional activators, some of the previously silenced genes can start transcription (activatable genes). Silencing of such genes is likely due to the absence of activators and/or the presence of repressors and hence addition of activators allows transcriptional activation from those genes. In other words, activators have access to those genes to induce gene activation. In contrast, some genes are not activated even if known activators of these genes are present (occluded genes). This is probably because occluded genes are silenced by chromatin-based mechanisms that preclude access of activators to target genes. It seems to take a longer time for activators to finally gain access to such genes. This classification of genes in response to transcriptional activators has been proposed by Lahn and colleagues [26, 27]. Activation from occluded genes can be enhanced by adding chromatin remodeling factors and chromatin modifiers that can relieve these chromatin-based repression mechanisms

Transcription from previously silenced genes can be induced by introduction of transcriptional activators (Fig. 1). In a special type of experimental design, nuclear gene expression of one kind of cell is switched to that of an embryo or other cell type, referred to as transcriptional reprogramming. Transcriptional reprogramming is achieved by different approaches, such as induced pluripotency, cell fusion, and nuclear transfer to eggs/oocytes [20]. However, it is generally accepted that the efficiency of transcriptional reprogramming is low [21]. This is due to the fact that gene expression is often repressed by layers of silencing mechanisms to maintain transcriptionally quiescent states [22]. Gene silencing seems to be progressively more difficult to reverse as cells become increasingly differentiated. This is exemplified by sequential epigenetic modifications for *Oct4* silencing during ES cell differentiation [23, 24] and differential gene reactivation from the inactive X chromosome between epiblast stem cells and differentiated mouse embryonic fibroblasts [25]. Moreover, mechanisms and extents of gene silencing are different depending on each gene. Lahn and his colleagues [26, 27] have proposed that genes that are not expressed can be classified into two categories based on resistance to transcriptional activation during cell-fusion-induced reprogramming (Fig. 1); (1) genes that are silent in one cell type are expressed (activatable) when that cell is fused with another cell type in which that gene is already active, and (2) genes that are silent in one cell type remain inactive (occluded) when that cell is fused to another cell type in which that gene is active [26, 27]. The former activatable genes are not expressed because of the absence of transcriptional activators and/or the presence of repressors and introduction of activators is enough to induce transcription. The latter occluded genes are proposed to be inactive due to chromatin-based repression mechanisms that maintain silent states regardless of whether transcriptional activators are present. Full activation of occluded genes normally requires multiple cell divisions and a longer time exposure to a cellular milieu that supports transcription from occluded genes than activatable genes, implying that de-repression of chromatin-based inhibition proceeds gradually. Although the classification has been carried out in the context of cell-fusion-mediated reprogramming, this concept seems to be generally applicable to other reprogramming systems and cellular events. For example, addition of retinoic acid (RA) to cells can cause rapid induction of transcription from RA-responding genes such as *Hox* genes. These transcribed genes are most likely activatable ones since they can respond to activators (RA receptor in this case). On the other hand, in iPS experiments, expression of many embryonic genes from somatic cells requires several days and cell divisions, although known transcriptional activators of these embryonic genes are highly expressed in the somatic cells transduced [28, 29]. This result argues that silenced embryonic genes in somatic cells are likely to be occluded genes. It is important to know the states of gene silencing when we study gene activation and transcriptional reprogramming.

## Factors involved in gene activation

Reprogramming experiments have revealed that resistance to transcriptional activation differs in different genes. They also bring up an intriguing question of what kinds of factors can overcome this resistance. In activatable genes, transcriptional activators are enough to rapidly induce transcription. This also suggests that those genes need to be accessible to activators. In other words, genes may have permissive chromatin states. Permissive chromatin is characterized by the presence of active histone marks such as histone acetylation, and the absence of repressive marks such as histone H3 lysine 9 (H3K9) methylation and DNA methylation [30]. Alternatively, it can be free from nucleosome occupancy [31, 32]. It is also possible that activatable genes are bound by paused RNA polymerase II (Pol II) [33] and introduction of activators enhances the transcriptional elongation step from paused Pol II. In fact, Pol II pausing is seen in many developmentally important genes for quickly achieving gene activation since these genes tend to be occluded by nucleosome formation without paused Pol II [34]. Thus, Pol II itself can work as a factor that precludes gene occlusion. Once occlusion happens, additional factors are necessary to induce transcriptional activation from such genes. In occluded genes, activators cannot access DNA-binding sites, such that RNA polymerase cannot be recruited to these genes. Heterochromatin formation may prevent access of activators. It is often associated with repressive marks such as DNA methylation, histone H3K9 di- or trimethylation, and repressors such as histone deacetylases and heterochromatin proteins. Reprogramming experiments have shown that these repressive modifications are removed before transcriptional reprogramming [35–37] and that removal of these modifications prior to reprogramming enhances its efficiency [24, 38, 39]. These results suggest that repressive modifications leading to compacted chromatin impede gene activation. It is hence plausible that addition of chromatin modifiers that can remove these repressive modifications is effective in reprogramming silenced genes [40–44]. Moreover, activation of silenced genes during reprogramming is enhanced by a chromatin remodeling factor, BAF complex [45], and addition of* trans*-activating domains to transcription factors [46, 47]. These factors allow efficient access for transcriptional activators to target genes. In conclusion, expression from occluded genes can be achieved by opening chromatin, in addition to providing transcriptional activators.

## Nuclear actin in transcriptional regulation

**Fig. 2:**
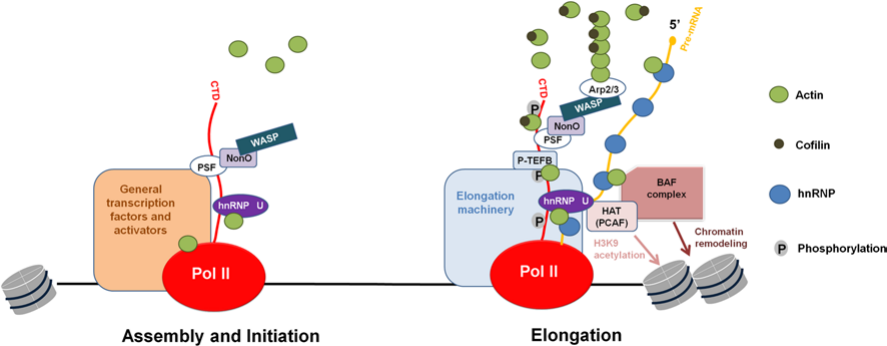
RNA polymerase II transcription is regulated by nuclear actin and actin-binding proteins. A model of nuclear actin- and actin-binding protein-mediated transcription by RNA polymerase II. Nuclear actin has been shown to interact with many proteins that play crucial roles in Pol II-mediated transcription. Actin directly interacts with Pol II and is required for the pre-initiation complex formation. PSF forms a complex with NonO and N-WASP and this complex can bind to the Pol II C-terminal repeat domain (CTD). During Pol II elongation, actin is necessary to mediate the association of P-TEFb and elongating Pol II (Serine 2 phosphorylated CTD Pol II). Actin and hnRNP U are associated with the hyperphosphorylated form of Pol II CTD and play an important role in recruiting histone acetyltransferases (HATs), which facilitate permissive chromatin states for transcription by acetylating histone H3K9. Actin also binds to hnRNP and hnRNP U on pre-mRNA. The BAF complex can associate with nascent pre-mRNP (ribonucleoprotein complexes) [140] and may affect chromatin remodeling for transcription. Furthermore, the PSF-NonO-N-WASP(or WASP) complex can interact with elongating Pol II. N-WASP promotes actin nucleation with the Arp2/3 complex. Polymerized actin (non-canonical actin filaments) may be readily depolymerized by cofilin to provide monomeric actin for transcriptional elongation. In fact, cofilin is exclusively associated with gene coding regions, but not with promoters [99]. In addition, actin polymerization may help to increase a local concentration of actin near the transcription site. Pre-mRNA produced by Pol II is indicated as a* yellow line* and the CTD of Pol II as a* red line*

**Fig. 3:**
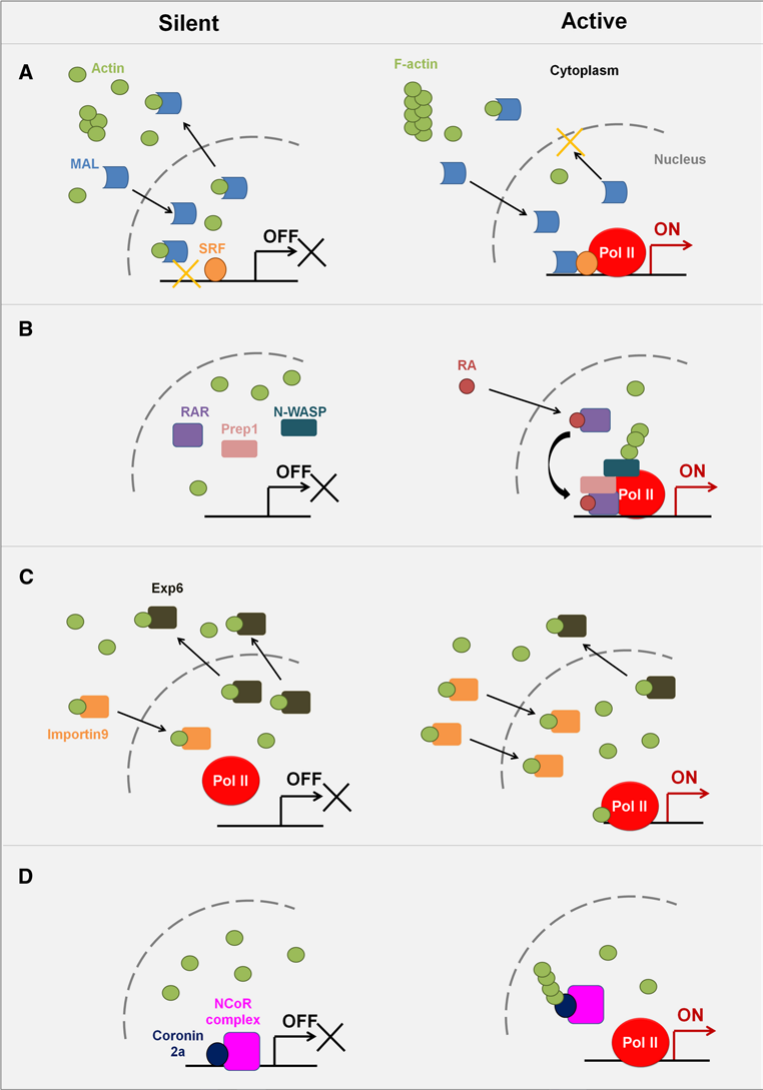
Nuclear actin and transcriptional activation. **a** Serum response factor (SRF) requires MAL, its coactivator, to achieve transcription from target genes. MAL translocates to nuclei, but is exported to the cytoplasm when it binds to monomeric actin, thus preventing transcriptional activation. When cytoplasmic actin is polymerized, the monomeric actin pool is decreased and hence MAL free from actin binding is increased, thereby inducing transcription from SRF target genes. **b** Retinoic acid (RA) activates the RA receptor (RAR) and RAR works as a transcriptional activator on its target genes together with Prep1 and N-WASP. N-WASP may recruit polymerized actin on active genes. **c** Nuclear actin levels are maintained by active nuclear import and export of actin. Importin 9 imports cytoplasmic actin to nuclei, while nuclear actin is exported to the cytoplasm by Exportin 6 (Exp6). High nuclear actin levels can support active transcription. **d** Nuclear co-repressor (NCoR) complexes inhibit transcription from Toll-like receptor-responsive genes. NCoR complexes contain Coronin 2A, which can bind to polymerized actin. Binding of actin polymers to Coronin 2A induces dissociation of NCoR complexes from silenced genes, thereby allowing transcriptional activation

Mechanisms of transcriptional activation and reprogramming have been described so far. A transcriptional activation process is affected by many nuclear events. Nuclear actin plays key roles in such events including basal transcription by all three RNA polymerases (Pol II case in Fig. 2) [48–51], chromatin remodeling [11, 52, 53], pre-mRNA processing [12, 54], and gene movement [55]. Moreover, recent studies indicate that nuclear actin is directly or indirectly involved in transcriptional activation (Fig. 3) [56–58]. Here, possible roles of nuclear actin in those cellular events are discussed.

### Nuclear actin in transcription

Involvement of nuclear actin in transcription was described for the first time in the early 1980s [59]. Injection of antibodies against actin into nuclei of amphibian oocytes induced retraction of lampbrush chromosomes, reminiscent of transcriptional cessation [59]. Recent studies using more sophisticated in vitro and in vivo transcription assays confirmed the concept of this early study. Actin is found in the preinitiation complexes (Fig. 2) and is important for transcriptional initiation by Pol II [49]. The phosphorylated C-terminal domain of Pol II is associated with actin [51]. Actin is also known to interact with the nascent transcripts [54] and heterogeneous ribonucleoproteins (hnRNPs) mediate binding of actin to the nascent RNAs [51]. hnRNPs and actin binding is necessary for transcription elongation by Pol II since a competing peptide that disrupts hnRNPs–actin interaction inhibited transcription elongation when examined by run-on assay [60]. The hnRNPs-actin interaction is important for the association of histone acetyltransferase PCAF and Ser2- and Ser2/5-phosphorylated Pol II [61]. This study [61], together with the work using *Chironomus tentans* [62], also brings a key concept that actin takes part in establishing permissive chromatin through recruitment of the histone acetyltransferase during transcription. Ser2 phosphorylation of Pol II is mediated by positive transcription elongation factor b (P-TEFb). Actin, especially monomeric actin (G-actin), facilitates recruitment of P-TEFb to Pol II [63], further affirming the importance of nuclear actin in transcription elongation. Collectively, nuclear actin is directly involved in RNA polymerase-mediated transcription through enhancing preinitiation complex formation and transcription elongation. One model of nuclear actin- and actin-binding proteins-mediated transcription is shown in Fig. 2 and our model is explained in the figure legend. Details about nuclear actin in transcription are discussed in recent insightful reviews [10, 12, 64].

### Nuclear actin in chromatin remodeling

Actin and actin-related proteins (Arps) have been found in many chromatin remodeling complexes [11, 16, 65, 66]. These remodelers play key roles in various nuclear events such as transcription, DNA repair, DNA replication, and nuclear organization. Since the functions of ARPs have been extensively reviewed [16, 65, 66], this paper discusses possible roles of actin in chromatin remodeling. An early discovery that nuclear actin participates in chromatin remodeling was made in the late 1990s. β-actin and ARP4 (also known as BAF53) are found in the mammalian SWI/SNF-like BAF chromatin remodeling complexes and tightly associate with the ATPase Brg1 in the complex [52]. The BAF complex requires actin for its full ATPase activity and its association with chromatin [52]. A recent study suggests that interaction between β-actin and ARP4 is important for the integrity of the BAF complex since interruption of heterocomplex formation by β-actin and ARP4 accelerates degradation of Brg1 [67]. The heterocomplex formation by G-actin and ARP4 is supported by biochemical studies [68]. Interestingly, Fenn et al. [68] has revealed that ARP4 is able to enhance depolymerization of actin filaments. This result brings up the possibility that polymerized states of nuclear actin may influence assembly or activity of the BAF complex. In fact, the BAF complex can bind to actin filaments [69]. It would be interesting to know the causal relationship between nuclear actin polymerization and activities of BAF complex. Another chromatin remodeling complex that contains actin and ARP4 is the INO80 complex. The catalytic ATPase subunits of both INO80 and BAF complexes contain the important domain for binding to ARPs and actin, referred to as the helicase-SANT-associated (HSA) domain [70]. In the INO80 complex, ARP4, ARP8, and actin are bound to the HSA domain in the core ATPase INO80 and ARPs directly interact with histones so that the INO80 complex can gain access to nucleosomes [71]. Thus, important progress has been made to decipher the roles of nuclear actin in chromatin remodeling. Interdisciplinary approaches using structural biology and molecular cell biology will accelerate our understanding of nuclear actin in chromatin remodeling.

### Nuclear actin in transcriptional activation

The implication of nuclear actin in basal transcription has been demonstrated. Interestingly, transcriptional regulation by actin is not limited to polymerase-mediated transcription itself. There seem to be several different mechanisms to regulate transcriptional activation. A first clear example of participation of nuclear actin in gene activation has been demonstrated by the Treisman group. Activation of serum response factor (SRF), a transcription factor that regulates many serum-inducible and muscle-specific genes, coincides with F-actin accumulation in cells [72]. Studies using actin mutants that do not polymerize or enhance polymerization revealed that G-actin is the regulator of SRF activity and activation of MAL, a coactivator of the SRF transcription factor [73, 74]. G-actin binds to MAL in nuclei. This actin binding inhibits MAL’s function as a transcription activator and enhances export of MAL to the cytoplasm in a serum-starved condition [56]. After serum stimulation, F-actin is accumulated in the cytoplasm and this should lead to a decreased G-actin pool, thereby increasing active MAL free from G-actin binding. Thus, nuclear actin, in concert with cytoplasmic actin, regulates transcriptional activation by sequestering an activator (Fig. 3a). This transcriptional regulation by actin polymerization-mediated control of the nucleo-cytoplasmic distribution of transcription regulators does not seem limited to MAL’s case [75, 76]. Since many signaling molecules affect cytoplasmic actin polymerization, this sort of an indirect effect of actin on gene transcription through sequestration might be seen in other signaling pathways.

Secondly, nuclear actin is involved in induction of *HoxB* transcription by retinoic acid (RA) treatment [57]. It is known that RA receptors are important for expression of *Hox* gene and *HoxB* expression depends on a Prep1–Pbx1 complex that works as a transcription activator [77]. Proteomic analysis revealed that Prep1 binds to Pol II and nuclear β-actin [78]. Prep1 also interacts with nuclear N-WASP (neuronal Wiskott–Aldrich Syndrome Protein) that enhances actin polymerization [57]. Depolymerization of actin, N-WASP knockdown, and overexpression of an actin mutant that does not polymerize inhibit *HoxB* transcription after RA treatment. Interestingly, chromatin immunoprecipitation (ChIP) analysis has revealed that elongating Pol II, Prep1, actin, and N-WASP are recruited to the *HoxB* enhancer in an actin polymerization-dependent manner. These results suggest that actin polymerization is required for *HoxB* gene activation possibly by mediating recruitment of the transcription complex to the regulatory region. It would be interesting to examine whether recruitment of the transcription complex to the *HoxB* coding region is similarly regulated by actin polymerization and whether N-WASP-mediated actin polymerization or other nuclear actin polymerization is critical for this recruitment. Therefore, nuclear actin seems important for recruiting active transcription machineries during gene activation (Fig. 3b).

Another interesting phenomenon during gene activation is translocation and accumulation of cytoplasmic actin to nuclei [19]. Actin dynamically shuttles between the nucleus and cytoplasm. Actin export from nuclei is accomplished by Exportin 6 [79]. However, it has been elusive whether cytoplasmic actin is actively imported into nuclei, although we know that cofilin is important for actin import in special circumstances, such as stress. Recently, importin 9 has been identified as a key molecule that actively imports cytoplasmic actin to nuclei possibly with cytoplasmic cofilin [80]. Moreover, this active maintenance of nuclear actin by importin 9 is necessary for maximal transcriptional activity for cells [80]. In accordance with this report, translocation of cytoplasmic β-actin to nuclei is observed during differentiation of human promyelocytic leukemia (HL-60) cells towards macrophages; this entails activation of many genes for successful differentiation [19]. During differentiation, association of nuclear actin with Pol II is observed. ChIP-on-chip assays revealed a striking increase of nuclear actin binding to gene promoters (25 to 827 genes). Knockdown of β-actin inhibits Pol II binding to promoters, suggesting that nuclear translocation of actin during differentiation allows efficient recruitment of Pol II to target genes (Fig. 3c). Interestingly, when cells become quiescent, nuclear β-actin is depleted and Pol II binding to transcription sites is destabilized [81]. This report further supports the idea that nuclear actin levels are an important determinant of transcriptional activity.

As mentioned above, nuclear actin has been identified in many chromatin remodeling complexes and chromatin modifiers. One study shows involvement of oligomeric actin in chromatin remodeling and gene activation [58]. Transcription from Toll-like receptor (TLR)-responsive genes is inhibited by nuclear receptor co-repressor (NCoR) complexes and clearance of this NCoR complex from promoters is necessary for transcriptional activation from TLR target genes. The NCoR complex includes Coronin 2A, an actin filament-binding protein. Nuclear actin binds to Coronin 2A and this binding triggers clearance of repressive NCoR complexes from the promoters of target genes, thereby inducing transcriptional activation. These results suggest that transcriptional activation by nuclear actin is achieved by removing gene silencing complexes from chromatin (Fig. 3d).

## Nuclear actin-binding proteins in transcriptional regulation

Nuclear actin-binding proteins as well as nuclear actin play a crucial role in transcription and transcriptional activation. One of the most studied nuclear actin-binding proteins is an isoform of myosin I (nuclear myosin I; NMI) [82, 83]. NMI is involved in transcription by RNA polymerase I [48, 84, 85] and II [86] and interacts with the chromatin remodeling complex WSTF-SNF2h [87]. Many other nuclear myosins have been identified and their diverse nuclear roles have been found [10]. Since functions of nuclear myosins are extensively summarized in recent reviews [10, 12, 88], this review will focus on other actin-binding proteins that can be involved in transcription and transcriptional regulation.

Members of the Wiskott–Aldrich syndrome protein (WASP) family are key factors of actin polymerization by regulating the actin-related protein 2/3 (Arp2/3) complex, an actin nucleator [89–91]. The WASP family consists of two classes of proteins; WASPs (WASP and N-WASP) and WAVEs (WAVE1, WAVE2, and WAVE3). Mutant WASP was identified as the causative gene of the Wiskott–Aldrich syndrome and WASP is expressed in hematopoietic cells while N-WASP is ubiquitously expressed. Although the WASP family proteins were originally identified as cytoplasmic proteins that regulate cortical actin filaments, nuclear WASPs and WAVEs have also been detected [17, 92–94]. N-WASP forms a nuclear protein complex containing non-Pou-domain octamer-binding protein (NonO), polypyrimidine-tract-binding-protein-associated splicing factor (PSF) and Pol II in human 293T cells [93]. Moreover, nuclear N-WASP regulates Pol II-mediated transcription through its interaction with NonO, as well as through the induction of actin polymerization. Interestingly, NonO has been shown to bind to hyperphosphorylated Pol II [95] and N-WASP can also be associated with elongating Pol II [57]. Therefore, it is tempting to speculate that N-WASP plays a role in the elongating step of transcription (Fig. 2). Since N-WASP is also found on gene promoters and binds to unphosphorylated Pol II [93], it is possible that N-WASP is also involved in transcriptional initiation or recruitment. In hematopoietic cells, WASP is expressed instead of N-WASP. WASP translocates into nuclei during T cell differentiation [17]. Nuclear WASP binds to histone modifying enzymes such as RBBP5, a histone H3K4 tri-methyltransferase, and JMJD2A, a H3K9/H3K36 tridemethylase, and RNA Pol II. WASP also binds to the transcription factor SP1. This SP1 binding seems important for recruiting WASP to specific target genes for transcriptional activation. Collectively, WASP plays an important role in transcriptional activation of genes required for T cell differentiation by regulating active histone marks and possibly transcription. Moreover, the actin nucleating Arp2/3 complex is found in nuclei and is required for a full Pol II activity [96]. Arp2/3 and F-actin bind to the gene regions to which WASP is bound [17]. The WASP family proteins have an ability to mediate various protein–protein interactions and this characteristic may help them to associate with many nuclear proteins and nuclear actin.

Cofilin/ADF (actin depolymerizing factor) enhances depolymerization of actin by severing F-actin [97]. Cofilin binds to G-actin and forms a heterodimer. This actin-cofilin complex is imported into nuclei [80, 98]. Cofilin-1 in nuclei seems to bind to the elongating type of RNA polymerase II and actin [99]. ChIP analysis revealed that cofilin-1 is associated with gene coding regions, but not with the promoter. Since G-actin is important for transcriptional elongation [63], cofilin-1 may provide a G-actin pool for Pol II elongation. Considering the cofilin’s F-actin severing function, actin polymers may exist adjacent to transcribing genes to provide enough cofilin-1 enrichment. Such polymeric actin might be produced by N-WASP and Arp2/3 functions and further accelerated by profilin (Fig. 2). The regulation of nuclear actin polymerization with the help of cofilin and profilin during transcription is summarized in [64].

A list of other nuclear actin-binding proteins is summarized in [14, 15, 100, 101]. The number of nuclear actin-binding proteins is likely to expand in the future [102, 103]. It is therefore important to define the relationship among these proteins. Most of the actin-binding proteins listed here are connected in the context of transcription and actin polymerization. Such classification will help to get a better view of the nuclear actin network and possibly nucleoskeleton [88]. It is also important to bear in mind that actin-binding proteins can have actin-independent roles in nuclei. This is exemplified by the profilin case, where profilin and actin seem to be recruited differentially to chromosomes [104]. We have recently found another such case in which a nuclear actin-binding protein can bind to the transcription apparatus without an actin-binding domain (unpublished data).

## Nuclear actin is needed for transcriptional reprogramming in *Xenopus* oocytes

**Fig. 4:**
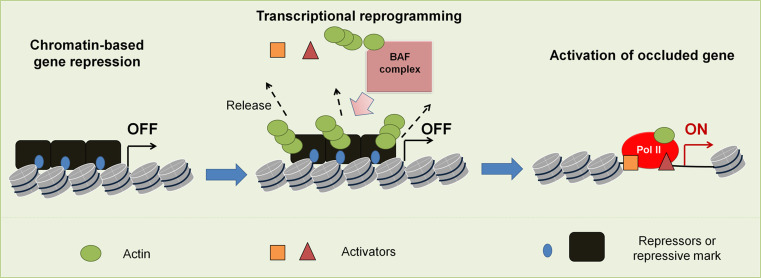
A model of nuclear actin-mediated transcriptional reprogramming of occluded genes. Expression of occluded genes, such as *Oct4*, is repressed by chromatin-based mechanisms. During transcriptional reprogramming, repressors and/or repressive marks that restrict access of transcriptional activators to chromatin may be removed with the help of nuclear actin. In addition, actin-containing chromatin remodeling complexes like the BAF complex can accelerate chromatin opening. Clearing chromatin-based repression enables transcriptional activators and Pol II to have access to promoters of occluded genes. Pol II-mediated transcription is also enhanced by nuclear actin

Nuclear actin serves as an important factor for gene activation by several different mechanisms. Most of the cases discussed so far deal with transcriptional activation from activatable genes, but not from occluded genes. This is because introduction of transcriptional activators is enough to induce transcription in these cases and nuclear actin functions in this context. Being different from these examples, transcriptional reprogramming entails activation of occluded genes. Transcriptional reprogramming can be induced in a direct and rapid manner by transplanting somatic nuclei into the nucleus of a *Xenopus* oocyte called the germinal vesicle (GV) [105]. Reprogramming in eggs/oocytes is proposed to be the most efficient way to induce pluripotency in somatic nuclei [105, 106] and activation of *Oct4*, an embryonic gene that was previously identified as an occluded gene in somatic cells in cell fusion experiments [26], is induced as early as 20 h after nuclear transfer as judged by the reporter expression [107]. In agreement with this, transcription from many embryonic genes including *Oct4*, *Sox2*, and *Nanog* is induced within a few days in somatic nuclei transplanted into *Xenopus* oocytes [108]. Thus, oocytes seem to have an ability to reprogram occluded genes that were defined in somatic–somatic or even somatic–ES cell fusion experiments [26]. Nuclear transfer to oocytes therefore provides an opportunity to evaluate unique oocyte factors that can overcome gene occlusion. *Xenopus* oocytes contain enormous amounts of actin in nuclei [109–111] and this nuclear actin has been linked to transcription from the oocyte genome [59]. Moreover, nuclear actin is found in transplanted nuclei [18]. Interestingly, polymerized actin is formed in nuclei transplanted to *Xenopus* oocytes, resembling nuclear actin seen in the GV of oocytes [18]. Disturbance of this nuclear actin polymerization impairs activation of *Oct4* transcription in transplanted nuclei, suggesting that nuclear actin polymerization is important for transcriptional activation of *Oct4*. Thus, nuclear actin seems involved in reversal of an occluded gene (Fig. 4), but we still do not know which step during reversal of silencing is caused by nuclear actin. One possibility is that nuclear actin accelerates chromatin remodeling by changing its polymerized states (Fig. 4). In fact, enhanced binding of actin to *Oct4* by overexpression of actin-binding protein Toca-1 coincides with enhanced BAF complex binding to *Oct4* during reprogramming [18]. It is also possible that actin polymers play a role in the clearance of transcriptional repressors as is the case of the NCoR complex (Fig. 4) [58]. We also ought to take into account the possibility that nuclear actin enhances recruitment or elongation of Pol II.

## Involvement of nuclear actin and actin-binding proteins in nuclear reprogramming

Possible participation of nuclear actin and actin-binding proteins in nuclear reprogramming has been suggested not only in the *Xenopus* nuclear transfer system, but also in other reprogramming systems. Firstly, actin-containing BAF remodeling complex is crucial for embryonic gene activation during reprogramming by the iPS route and egg extract treatment [45, 112] and is proposed to convert inaccessible chromatin of embryonic genes in somatic cells to accessible states for transcriptional activators [45]. It would be interesting to examine the function of nuclear actin in concert with the BAF complex during reprogramming. Secondly, Polycomb repressive complex (PRC) is well known as a chromatin remodeler that silences developmentally important genes such as *Hox* genes and is essential for normal development. It is also necessary for reprogramming somatic nuclei towards pluripotency in cell-fusion and iPS experiments [113, 114]. Ezh2 is a catalytic subunit of PRC2. Interestingly, Ezh2 is also present in the cytoplasm and regulates cytoplasmic actin polymerization [115]. Although it is not clear whether Ezh2 regulates nuclear actin polymerization in a similar manner, PRC is an interesting candidate for examining its relation to nuclear actin. Thirdly, the translationally controlled tumor protein (TCTP) is involved in regulating many cellular processes such as cell proliferation and apoptosis and is necessary for development. TCTP has been shown to enhance activation of pluripotency genes including *Oct4* in transplanted nuclei into *Xenopus* oocytes [116]. TCTP is also implicated in nuclear reprogramming in bovine oocytes although the mechanisms are still unknown [117]. Intriguingly, TCTP contains cofilin-like actin-binding site and binds to G-actin [118] and *Xenopus* TCTP is also associated with F-actin [119]. The observed positive effects of TCTP on transcriptional reprogramming therefore might be through altering nuclear actin polymerization. Lastly, actin in mammalian oocytes seems to have a great impact on reprogramming. Incorporation of oocyte actin into somatic nuclei is observed during incubation in porcine oocyte extracts [120, 121], which are known to induce a part of early reprogramming events [122]. This implies that actin is likely to be involved in an early step of reprogramming in oocytes. In accordance with this idea, different groups have recently reported that treatment of nuclear transferred embryos with actin depolymerizing reagents greatly affects development of these embryos; Latrunculin A, instead of Cytochalasin B, significantly improves cloning efficiency [123–125]. It is unclear whether this positive effect is caused just by reduced cytotoxicity of the actin depolymerizing reagent used or by improved some reprogramming aspects related to actin polymerization states. Answering this question may advance our understanding on nuclear actin in reprogramming.

## Mechanistic insight into how nuclear actin plays a role in nuclear reprogramming

Several studies have thus implied that nuclear actin is implicated in nuclear reprogramming. However, functions of nuclear actin in reprogramming are obscure. In this section, we propose some speculative roles of nuclear actin during reprogramming. When we define nuclear reprogramming as a phenomenon in which differentiated cells are reversed to an embryonic state, reprogramming is composed of several distinct processes, such as extinction of differentiation gene expression, initiation and continuous expression of embryonic genes, and establishment of embryonic cell properties including high DNA repair activities, high telomerase activities, and embryonic cell-specific nuclear architectures, etc. One plausible role of nuclear actin in the reprogramming process is that actin is required for transcription of embryonic genes. Many embryonic genes need to be activated and continuously expressed during reprogramming. Nuclear actin might support efficient transcription from those genes by accelerating Pol II initiation and elongation. Also, nuclear actin-mediated chromatin remodeling might be important for reprogramming. Nuclear reprogramming entails activation of occluded genes, which are silenced due to the chromatin-based repression mechanisms (Fig. 4). The BAF, INO80, and Tip60 chromatin remodeling complexes that contain actin can help to establish open or unstable chromatin states. Nuclear actin may affect the activities of such remodeling complexes to induce transcription from occluded genes (Fig. 4). Therefore, it is interesting to investigate the binding of nuclear actin and such remodeling complexes to embryonic genes during reprogramming. In fact, the BAF and Tip60 complexes have been shown to maintain pluripotency by regulating gene expression in ES cells [126–128]. It is also noteworthy that reprogramming towards an embryonic state is accompanied not only by activation of embryonic genes but also by repression of differentiation genes. A recent study, in which β-actin knockout cells are used, showed that β-actin seems to have both gene-activating and gene-repressing activities [129]. Although this idea needs to be further tested, the new actin’s function in repressing genes can be explained in concert with chromatin remodeling complexes. The actin-containing BAF complex also plays a role in silencing genes in ES cells [126]. Therefore, nuclear actin might be involved in silencing differentiation genes through chromatin remodeling during reprogramming.

Apart from roles of nuclear actin in transcription, nuclear actin-mediated DNA repair might be involved in the reprogramming process. Embryonic cells and iPS cells possess high DNA repair capacities and DNA repair pathways have to be active for successful reprogramming in the iPS route [130]. Moreover, DNA repair events are likely to be induced in nuclear transfer embryos [131]. A recent study has shown that polymeric actin is required for proper DNA double-strand break repair [132]. Together, it may be worth pursuing the relationship between nuclear actin polymerization and DNA repair during reprogramming and in reprogrammed embryonic cells.

Importantly, we also need to take account of the fact that actin may affect mechanical properties of the nucleoskeleton. Some nucleoskeleton structures are cell type-specific, such that metastatic cancer cells exhibit abnormal chromatin organization [88]. Embryonic cells, as well as reprogrammed cells, seem to possess a specific nucleoskeleton structure since they have a different lamin composition from differentiated cells [133, 134] and exhibit a different chromocenter compartment [135]. Changes of nucleoskeleton organization can influence expression of a large number of genes (>1,000 genes) [136] and hence establishment of a proper nucleoskeleton is important. Actin may coordinate nucleoskeleton organization processes through interactions with its many binding partners in nuclei. Interesting insights might be gained by examining the effects of altering actin polymerization on chromatin reorganization during reprogramming.

As mentioned above, multiple steps are required for reprogramming somatic cells. Nuclear actin seems to play a role in many steps during reprogramming; this argues that actin might be an important player in reprogramming. This is attributed to multifunctional properties of nuclear actin. Such properties of actin probably result from the fact that it has a myriad of binding partners and can affect activities of its binding molecules. To reveal how nuclear actin participates in each step of reprogramming will expand our understanding of reprogramming and nuclear actin biology.

## Conclusions and perspectives

Transcriptional activation is a fundamental cellular process essential for living organisms. Transcription can be induced by adding activators such as transcription factors. However, in some cases, genes are highly silenced (occluded) with layers of repressing mechanisms, which cannot be easily overcome by activators due to the lack of access to these silenced genes, requiring transcriptional reprogramming that includes de-repression of silenced chromatin states. Nuclear actin seems to play important roles in transcriptional activation from both activatable and occluded genes. This intriguing feature of nuclear actin may be attributed to its significant function in basal transcription and chromatin remodeling. Furthermore, nuclear actin and nuclear actin-binding proteins participate in other nuclear processes that potentially relate to transcriptional activation and reprogramming such as nucleoskeletal activities [88] and gene movement upon activation [55, 137, 138]. Recent studies have shown that more and more proteins that regulate actin dynamics are found in nuclei [102, 103]; these imply that our understanding of the functions ascribed to nuclear actin may be extended. For obtaining a global view of the relationship between nuclear actin and actin-binding proteins, it would be interesting to investigate the association of actin and actin-binding proteins with genes in a genome-wide level by ChIP-seq analysis. A genome-wide study using *Drosophila* cells has shown that actin is associated with active euchromatin regions [139], in agreement with the general view that actin works as a positive regulator of transcription. Examining the genome-wide binding of nuclear actin and actin-binding proteins in different cell types and different conditions such as during differentiation will help us to achieve an overview of actin-mediated regulation of gene transcription. This may also answer the important question as to how gene expression is regulated by nuclear actin in a gene-specific manner. Nuclear actin is not just a byproduct of abundant cytoplasmic actin. A better understanding of nuclear actin in transcriptional activation and reprogramming will lead on to revealing its unknown functions in cellular and developmental contexts.
